# Impact of long-term optimizing atrioventricular delay using device-based algorithms on cardiac resynchronization therapy

**DOI:** 10.1007/s00380-022-02162-4

**Published:** 2022-09-29

**Authors:** Yoshifumi Ikeda, Ritsushi Kato, Kenta Tsutsui, Hitoshi Mori, Daisuke Kawano, Sayaka Tanaka, Shintaro Nakano, Takaaki Senbonmatsu, Shiro Iwanaga, Toshihiro Muramatsu, Kazuo Matsumoto

**Affiliations:** 1 Department of Cardiology, Saitama Medical University International Medical Center, 1397-1 Yamane, Hidaka, Saitama 350-1298 Japan; 2 Department, Research Administration Center, Saitama Medical University, Saitama, Japan

**Keywords:** Atrioventricular delay, Cardiac resynchronization therapy, Device-based algorithms, Heart failure

## Abstract

Sub-optimal atrioventricular delay (AVD) is one of the main causes of non-responder for cardiac resynchronization therapy (CRT). Recently, device-based algorithms (DBAs) that provide optimal AVD based on intracardiac electrograms, have been developed. However, their long-term effectiveness is still unknown. This study aims to investigate the effect of optimizing AVD using DBAs over a long period, on the prognosis of patients undergoing CRT. A total of 118 patients who underwent CRT at our hospital between April 2008 and March 2018, were retrospectively reviewed; 61 of them with optimizing AVD using DBAs were classified into the treated group (group 1), and the remaining 57 were classified into the control group (group 2). The median follow-up period was 46.0 months. The responder and survival rate in group 1 were significantly better than those in group 2 (group 1 vs. group 2: responder rate = 64% vs. 46%, *p* = 0.046; survival rate: 85.2% vs. 64.9%, *p* = 0.02). Moreover, investigating only the non-responder population showed that group 1 had an improved survival rate compared to group 2 (group 1 vs. group 2 = 72.7% vs. 45.1%, *p* = 0.02). Optimizing AVD using DBAs was a significant contributor to the improved survival rate in CRT non-responders in multivariate analysis (HR 3.6, *p* = 0.01). In conclusion, the long-term optimizing AVD using DBAs improved the survival rate in CRT and the prognosis of CRT non-responders, as well.

**Supplementary Information:**

The online version contains supplementary material available at 10.1007/s00380-022-02162-4.

## Introduction

Cardiac resynchronization therapy (CRT) was first applied clinically in the 1990s and is an important option for non-drug therapy in symptomatic heart failure with reduced ejection fraction (HFrEF) associated with cardiac dyssynchrony [1]. The prognosis of responders to CRT has been quite good and those patients have benefitted sufficiently from this therapeutic option [2, 3]. In contrast, the prognosis of non-responders to CRT has been dismal and it is still difficult to predict the non-responders to CRT before implantation, although CRT indication according to the guidelines has been strictly defined by several parameters, and it has been revised frequently. Currently, the class I indication for CRT in major international guidelines is patients with symptomatic HFrEF with sinus rhythm, complete left bundle branch block (CLBBB), wide QRS (over 150 ms), non-ischemic cardiomyopathy, and left ventricular ejection fraction (LVEF) less than 35% [4, 5]. In fact, the rate of non-response to CRT is still high (~ 30%) even after rigorous selection of patients. Mullen et al., reported that there were various suspected causes of non-response to CRT during follow-up [6]. According to this report, sub-optimal AVD was the most important reason for non-response to CRT, followed by arrhythmia, anemia, bi-ventricular (BiV) pacing rate, sub-optimal medical therapy, and so on. Transthoracic echocardiography (TTE)-guided approach is a useful method of AVD optimization. There are two major methods of estimating the optimal AVD, using TTE. One method is to estimate the cardiac output for various AVDs from the velocity time integral (VTI) using Doppler flow, and the other is to estimate the optimal AVD using the timing of two waveforms, that is, E (early diastolic) and A (atrial contraction) waves from the LV inflow pattern [7, 8]. However, those methods are time-consuming and relatively large intra- and inter-observer differences could arise. Currently, major manufacturers of cardiac implantable electric devices have developed device-based algorithms (DBAs) that calculate the optimal AVD and timing between the right and left ventricles (ventriculoventricular delay, VVD) based on intracardiac electrograms. Two categories of DBAs with CRT have been provided by manufacturers. The first is a manual type that is performed by a physician at the time of outpatient visits, and the second is an automatic type in which the device optimizes the AVD periodically (the timing depends on the algorithm). QuickOpt^™^ (Abbott) and SmartDelay^™^ (Boston Scientific) are examples of the former type, and SyncAV^™^ (Abbott) and AdaptiveCRT^™^ (Medtronic, as aCRT) are examples of the automatic type [9–12]. However, it remains controversial whether those algorithms can increase the number of CRT responders, and the effect of their long-term use on prognosis is unknown. Therefore, this study aims to clarify the influence of long-term optimization of AVD using DBAs, on the prognosis of patients undergoing CRT even though two different types of DBAs were used, i. e. the manual type and the automatic type.

## Materials and methods

### Patient selection

We retrospectively reviewed the records of 188 patients undergoing CRT, who were admitted to the International Medical Center, Saitama Medical University, Japan, between April 2008 and March 2018. The inclusion criteria were as follows; aged ≥ 20 years, provision of informed consent to undergo CRT at our hospital, and ≥ 6 months of follow-up at the outpatient clinic in our hospital. The exclusion criteria were as follows; refusal to participate in the study, an inability to optimize AVD due to chronic atrial fibrillation, patients with optimal AVD using TTE and those in whom it was difficult to continue AVD optimization for any reason, an inability to undergo follow-up at our hospital for any reason, and patients who turned off CRT within 1 year of implantation.

**Fig. 1:**
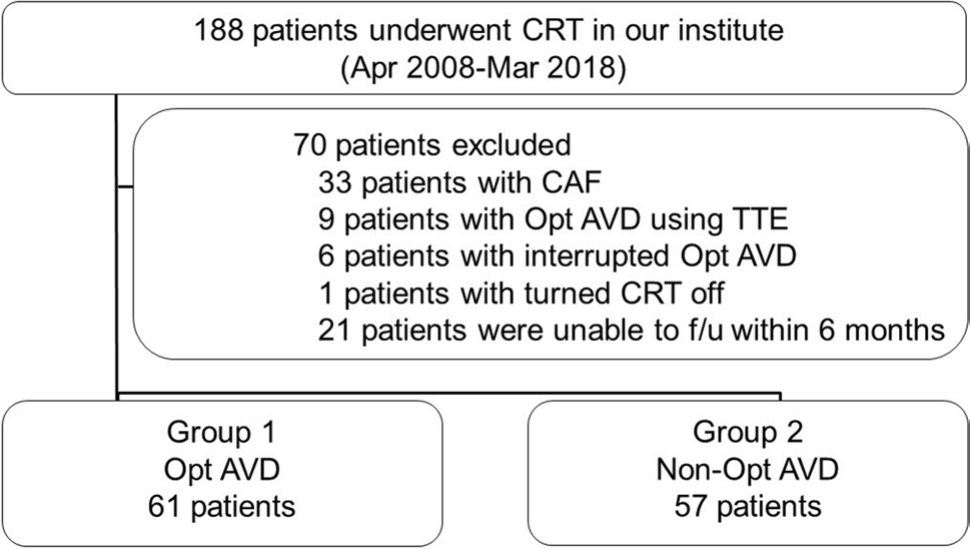
Patient selection. Flow diagram showing patient eligibility. *CRT* cardiac resynchronization therapy, *CAF* chronic atrial fibrillation, *AVD* atrio-ventricular delay, *TTE* transthoracic echocardiography; Opt, optimizing

In total, 118 patients were included in the analysis (Fig. 1). Sixty-one patients undergoing CRT with optimizing AVD using DBAs were categorized into the treated group (group 1) and 57 with non-optimizing AVD were categorized into the control group (group 2). In both groups, patients’ characteristics, etiologies, comorbidities, and medications were investigated, and the survival rates of both groups and their predictive factors were also analyzed. Response to CRT was assessed on LV remodeling (defined as a reduction in LV end-systolic volume of over 15% from the baseline) using TTE at 6 to 12 months after CRT implantation.

### Basic device setting (AVD and VVD)

The basic mode of the device was DDD, the minimum heart rate was set to 60 beats per minute (bpm), and the maximum heart rate was set to 130 bpm. The paced AVD was usually set to 180 ms, and the sensed AVD was set to 150 ms, if the device did not have DBAs. The AVD settings varied depending on the DBAs of each manufacturer. The details of DBAs are described as follows:

An algorithm named QuickOpt has been used from 2008 to the present in Abbott’s CRT [9]. QuickOpt is the first algorithm created for optimizing AVD in CRT using intracardiac EGM in CRT devices. AVD of QuickOpt timing cycle optimization is provided based on the *p* wave duration from the atrial lead and has been clinically proven to correlate with optimal AVD using echo-based optimization techniques. The algorithm was performed manually at every outpatient visit. This algorithm can also be used for patients with atrioventricular block (AVB). A new algorithm for AVD optimization called SyncAV has been used since 2016. If patients have their own AV conduction, we prefer SyncAV to QuickOpt [12]. Naturally, SyncAV cannot be used in patients with AVB. The SyncAV algorithm automatically extends the paced or sense AV delay (350 ms and 325 ms, respectively) by 3 beats after every 256 beats. Then, the optimal AVD is continuously set based on the measured self-AV conduction time. The AVD calculated using the SyncAV algorithm has been reported to correlate well with that calculated using QuickOpt. An algorithm named SmartDelay has been used in Boston’s devices since 2011. The SmartDelay algorithm is a DBAs that measures the optimal AVD from patients’ own AV conduction time, similar to the Sync AV algorithm. However, the algorithm must be performed manually at every outpatient visit. It is reported that the AVD determined from this algorithm correlates with the optimal AVD measured on echocardiography, although neither SmartDelay nor echocardiography was superior to a fixed AV delay of 120 ms [10]. AdaptiveCRT (aCRT) is a DBAs that periodically measures intrinsic conduction and dynamically adjusts CRT pacing parameters [11]. This algorithm has been available in Japan since 2014. The aCRT algorithm automatically extends the paced or sensed AV delay every minute and measures the conduction time from the right atrium to the right ventricle. If the conduction time is normal (AV ≤ 200 ms during sinus rhythm), the algorithm provides left LV-only pacing to make a fused QRS complex with its own right ventricular conduction. If the intrinsic AV conduction time is prolonged (more than 200 ms) during sinus rhythm or AVB, the algorithm provides BiV pacing. In those patients, the AVD and VVD are adjusted based on the length of the P and QRS waves, and their own AV conduction time, similar to QuickOpt. Therefore, aCRT is a DBAs that can be used in patients with AVB. The CRT of Biotronik has a DBAs named CRT AutoAdapt™, but patients in whom Biotronik’s CRT was implanted were categorized as group 2, because the algorithm was not available in Japan at the time of this study. Additionally, the Microport’s Respond-CRT™ was not used in this study [13]. In patients with automated DBAs, the optimal VVD was calculated automatically and simultaneously. In other patients, the optimal VVD was calculated using the delay between the RV and LV sensing during sinus rhythm or the delay between the sensing time of RV lead from the LV-only pacing and the sensing time of LV lead from RV-only pacing, if patients did not have their own AV conduction.

### Implantable cardioverter defibrillator (ICD) therapy setting

In primary prevention patients, ICD therapy was usually set at one zone for ventricular fibrillation (VF) only, and the detection rate was 200–220 bpm. We set three zones, that is, the monitor, ventricular tachycardia (VT) therapy, and VF zones for patients with secondary prevention. The VT therapy zone was customized according to the rate of each VT. The anti-tachycardia pacing (ATP) for VT was usually set at 5 to 8 burst pacing with approximately 90% R–R coupling interval of detected VT. The VF zone was set at approximately 200–220 bpm.

### Device follow-up

The patients were required to visit the device outpatient clinic at our hospital after CRT implantation. The performance of the devices was examined at 1 month and every 3 months thereafter, within the first post-implantation year, and it was subsequently examined every 6 months. A remote monitoring system (RMS) was applied to all patients from 2015, and if the RMS performance was stable, the follow-up period was prolonged to every 6 months from the first year of post-implantation period. Electrocardiography and chest radiography were performed at every visit. Echocardiography was performed at 6, 12, and 24 months after the implantation. It was also performed at the physicians’ discretion after the 2-year follow-up visit.

### Statistical analysis

Continuous variables were compared between the two groups using the Student's *t* test after confirming a normal distribution. Categorical variables were compared using the *χ*^2^ test. The Kaplan–Meier method was used to estimate the survival rate and the event-free rate, and the differences between the curves were compared using log-rank analysis. The error bars were used for comparisons that follow a normal distribution. The vertical bars indicated the range of standard deviation and central circle indicated mean value. Cox proportional hazards regression was performed using survival as the objective variable. Dependent variables were selected based on stronger factors for survival in univariate analysis, in addition to optimizing AVD using DBAs.

Statistical evaluations were performed and all statistical figures such as the error bars and Kaplan–Meier curves were created using SPSS version 21 software (IBM Corp., Armonk, NY, USA).

## Results

### Patient’s characteristics

The median follow-up period was 46.0 months, (range: 7–138 months). The baseline characteristics of patients in groups 1 and 2 are shown in Table 1. There was a significant difference in responder rate between two groups (group 1 vs. group 2: *n* = 39 (64%) vs. *n* = 26 (46%), *p* = 0.046), and the QRS width of group 1 was significantly wider than that of group 2 (166.1 ± 22.8 ms vs. 154.2 ± 30.1 ms, *p* = 0.02). The number of Abbott's CRT was significantly smaller in group 1 because Abbott’s QuickOpt was the oldest available algorithm. However, the number of Biotronik’s CRT was significantly large in group 2, because the algorithm of Biotronik for optimizing AVD could not be used during the observation period of this study. There were no significant differences between the two groups in other parameters of baseline characteristics.

**Table 1:** Patient’s characteristics

	Group 1			Group 2			*P* value
	(*n*=61)			(*n*=57)			
Basic information							
Male	64%	(*n*=39)		72%	(*n*=41)		NS
Responder	64%	(*n*=39)		46%	(*n*=26)		0.046
NICM	70%	(*n*=43)		77%	(*n*=44)		NS
ICM	28%	(*n*=18)		23%	(*n*=16)		NS
With defibrillator	89%	(*n*=54)		77%	(*n*=44)		NS
NYHA classification	2.9	±	0.57	3.0	±	0.71	NS
Age (y)	67.0	±	12.35	67.6	±	15.87	NS
Ht (cm)	159.9	±	8.72	161.8	±	9.33	NS
Wt (kg)	60.2	±	14.87	60.4	±	15.01	NS
BMI (kg/m^2^)	23.3	±	4.24	22.9	±	4.34	NS
Examination							
ECG							
CLBBB	75%	(*n*=46)		60%	(*n*=34)		NS
QRS width (msec)	166.1	±	22.84	154.2	±	30.13	0.02
TTE							
LVEF (%)	26.0	±	8.08	26.1	±	8.30	NS
LVDd (mm)	63.5	±	9.23	64.6	±	7.80	NS
LVESV (ml)	130.3	±	54.20	130.6	±	59.33	NS
MR	18%	(*n*=15)		21%	(*n*=19)		NS
PH	13%	(*n*=8)		16%	(*n*=9)		NS
Blood labor							
Cr (mg/dl)	1.3	±	1.14	1.2	±	0.45	NS
Na (mg/dl)	138.7	±	4.10	137.7	±	4.05	NS
Hb (g/dl)	12.7	±	2.02	13.4	±	1.82	NS
BNP (pg/ml)	733.0	±	862.44	814.7	±	764.31	NS
Comorbidity							
PAF	21%	(*n*=13)		23%	(*n*=13)		NS
VT/NSVT	41%	(*n*=25)		58%	(*n*=33)		NS
HT	34%	(*n*=21)		32%	(*n*=18)		NS
HL	31%	(*n*=19)		32%	(*n*=18)		NS
DM	25%	(*n*=15)		32%	(*n*=18)		NS
Device manufactures							
Abbott	57%	(*n*=35)		9%	(*n*=5)		<0.0001
QuickOpt	54%	(*n*=33)		NA			
SyncAV (1pt overlap)	5%	(*n*=3)		NA			
Boston scientific	20%	(*n*=12)		26%	(*n*=15)		NS
Medtronic	23%	(*n*=14)		46%	(*n*=26)		0.01
Biotronik	0%	(*n*=0)		19%	(*n*=11)		<0.0001
Median follow-up time			46.0 (7-138)			months	

*NICM* non-ischemic cardiomyopathy, *ICM* ischemic cardiomyopathy, *NYHA* New York Heart Association, *Ht* height, *Wt* weight, *BMI* body mass index, *ECG* electrocardiogram, *CLBBB* completely left branch block, *TTE* transthoracic echography, *LVEF* left ventricular ejection fraction, *LVDd* left ventricular diastolic diameter, *LVESV*: left ventricular end-systolic volume, *MR* mitral regurgitation, *PH* pulmonary hypertension, *Na* serum sodium concentration, *Cr* serum creatinine, *Hb* hemoglobin, *BNP* serum brain natriuretic peptide, *PAF* paroxysmal atrial fibrillation, *VT* ventricular tachycardia, *NSVT* non-sustained ventricular tachycardia, *HT* hypertension, *HL* hyperlipidemia, *DM* diabetes mellitus

The number in the brackets is the number of patients

The medications administered for HFrEF are listed in Table 2. The rate of amiodarone use was significantly lower in group 1 (group 1 vs. group 2: 36% vs. 58%, *p* = 0.02), but the rate of use of other drugs was similar between the groups.

**Table 2:** Medication used in both groups

	Group 1 (n=61)		Group 2 (n=57)		*P* value
Beta blocker	79%	(n=48)	77%	(n=44)	NS
ACE/ARB	79%	(n=48)	84%	(n=48)	NS
MRA	59%	(n=36)	68%	(n=39)	NS
OAC	39%	(n=24)	39%	(n=22)	NS
Anti-platelet	34%	(n=21)	40%	(n=23)	NS
Diuretics	72%	(n=44)	79%	(n=45)	NS
Amiodarone	36%	(n=22)	58%	(n=33)	0.02
Digoxin	5%	(n=3)	4%	(n=2)	NS

*ACE* angiotensin-converting enzyme inhibitor, *ARB* angiotensin receptor blocker, *BB*: *MRA* mineralocorticoid receptor antagonist, *OAC* oral anti-coagulation

The number in the brackets is the number of patients

### Comparison of survival rate between the responders and non-responders in both groups.

**Fig. 2:**
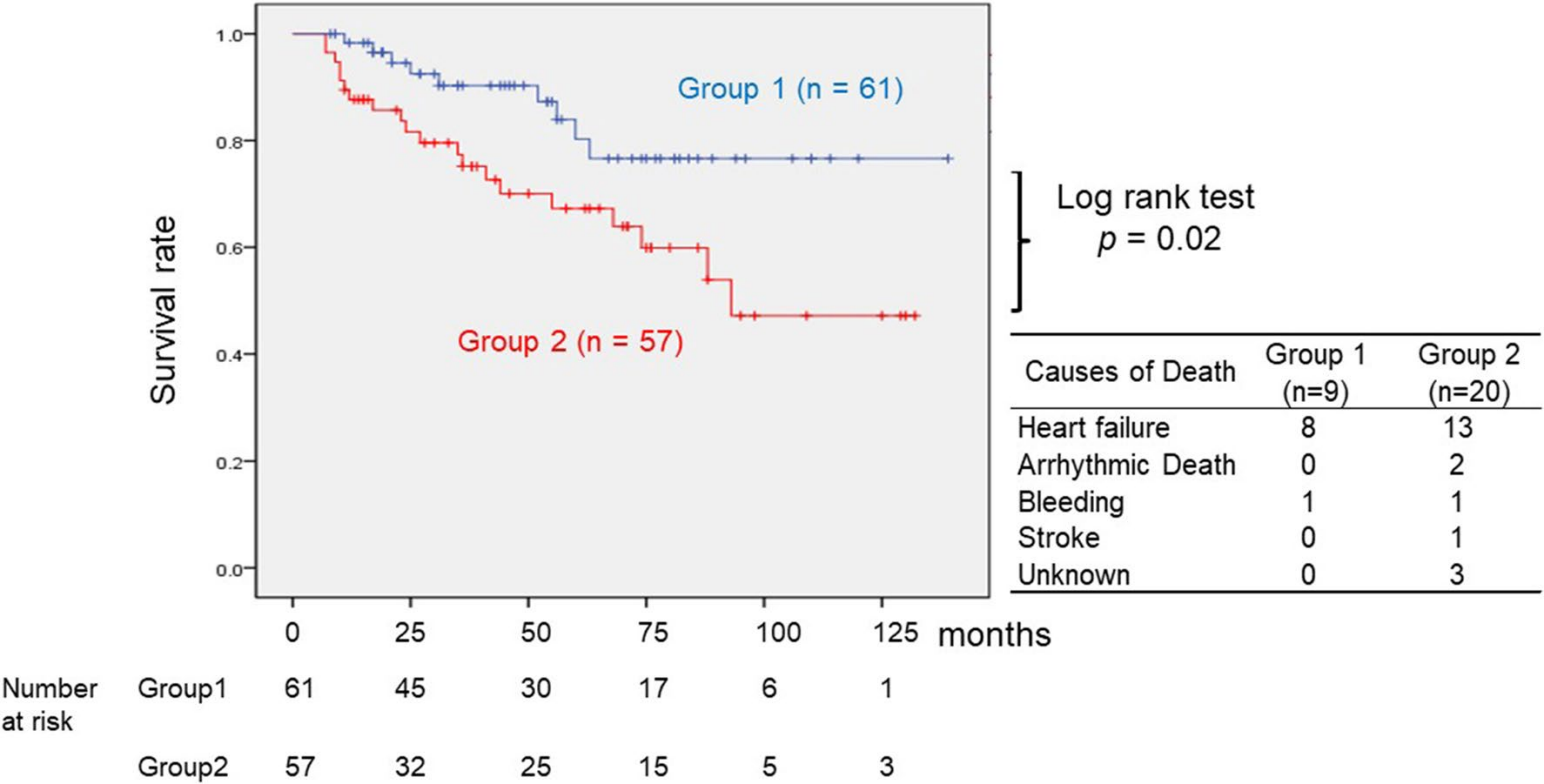
Comparison of survival rate between group 1 and 2 and causes of death. This shows the survival rate between two groups using the Kaplan–Meier method (blue line: group 1, red line: group 2). The survival rate of group 1 was higher than that of group 2; the deference was significant (*p* = 0.02). (Kaplan–Meier method was used to estimate survival rate, and differences between the curves were compared using log-rank analysis; *p* < 0.05 was considered statistically significant)

Figure 2 shows the comparison of 10-year survival (Kaplan–Meier curve) and causes of death between groups 1 and 2. According to the log-rank test, the survival rate of group 1 was significantly higher than that of group 2 (group 1 vs. group 2 = 52 / 61 vs 37 / 57 patients, log-rank test: *p* = 0.02).

The total number of deaths in group 1 and group 2 was 9 and 18. Heart failure was the main cause of death in both groups.

**Fig. 3:**
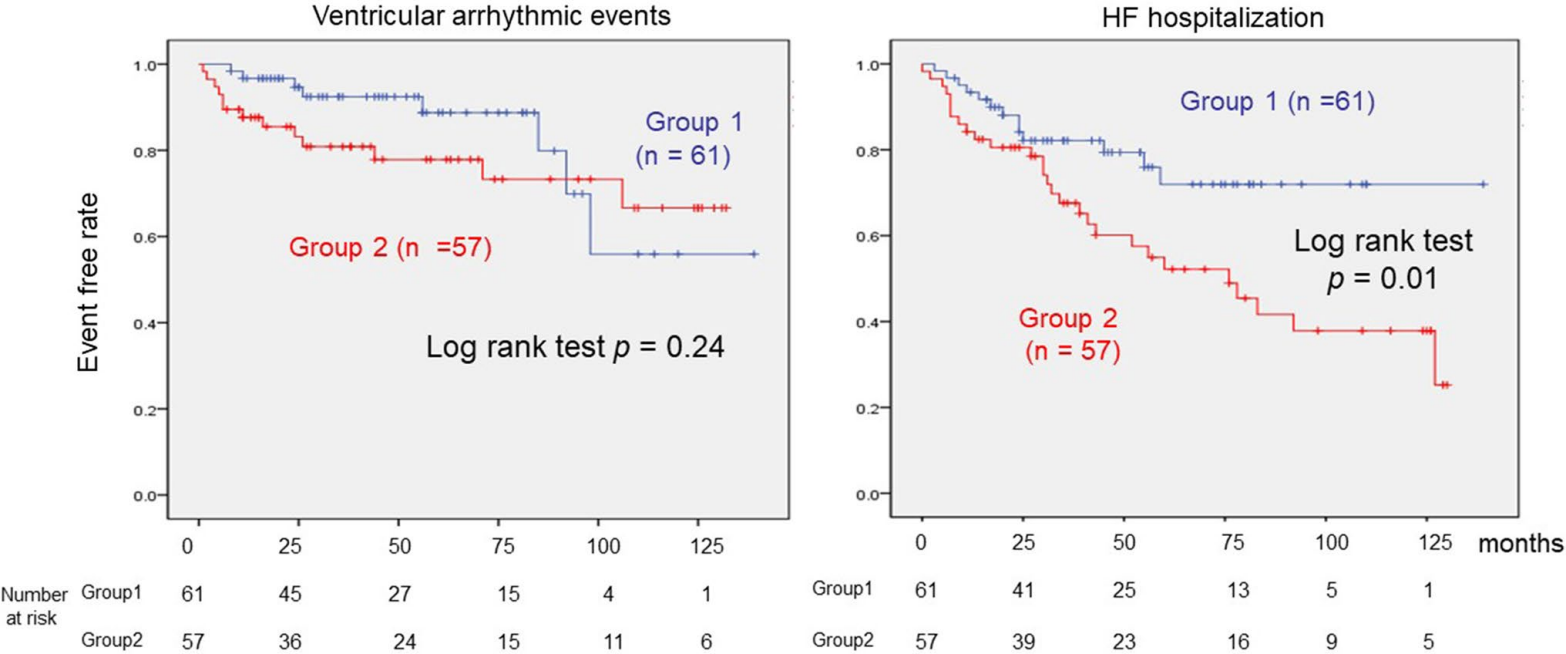
Comparison of ventricular arrhythmic events and HF hospitalization between groups 1 and 2. This shows the ventricular arrhythmic events (right side) and HF hospitalization (left side) between two groups using the Kaplan–Meier method (blue line: group 1, red line: group 2). The event-free rate of HF hospitalization of group 1 was higher than that of group 2; the deference was significant (*p* = 0.01), however, there was no significant difference for the ventricular arrhythmia events between the two groups. (Kaplan–Meier method was used to estimate survival rate, and differences between the curves were compared using log-rank analysis; *p* < 0.05 was considered statistically significant). *HF* heart failure

Figure 3 shows the comparison of ventricular arrhythmic events and HF hospitalization between groups 1 and 2 using the Kaplan–Meier curve. The ventricular arrhythmic events were defined as sustained ventricular tachycardia, ventricular fibrillation, and appropriate ICD therapy. There was no significant difference in event-free rate of ventricular arrhythmia between the groups (group 1 vs. group 2 = 53 / 61 vs. 44 / 57, log-rank test: *p* = 0.24) although the event-free rate of HF hospitalization in group 1 was significantly higher than that in group 2 (group 1 vs. group 2 = 48 / 61 vs. 29 / 57, log-rank test: *p* = 0.01).

**Fig. 4:**
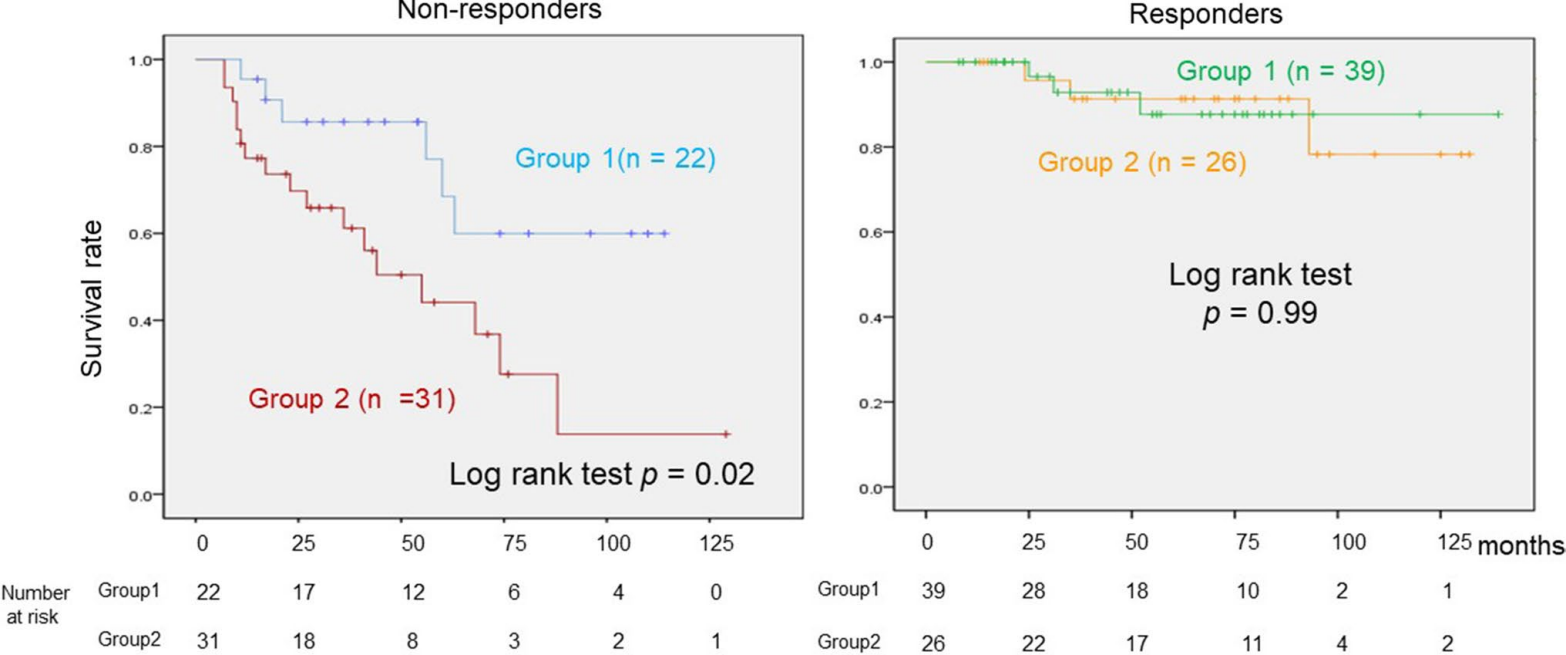
This shows a comparison of the survival rates of groups 1 and 2, focusing on responders and non-responders. The survival rate of non-responders in group 1 was higher than that of group 2; the difference was significant (*p* = 0.02), but there was no significant deference in the survival rates of responders in groups 1 and 2 (*p* = 0.99). Light blue line: non-responders in group 1, dark red line: non-responders in group 2, green line: responders in group 1, yellow line: responders in group 2

We then analyzed the survival rates of groups 1 and 2 in responders and non-responders, respectively. The survival rates of groups 1 and 2 were similar in the responders (log-rank test, *p* = 0.99), but the survival rate of group 2 in the non-responders was significantly lower than that of group 1 (group 1 vs. group 2 in non-responders = 16 / 22 patients vs. 14 / 31 patients, log-rank test: *p* = 0.02). (Fig. 4).

**Fig. 5:**
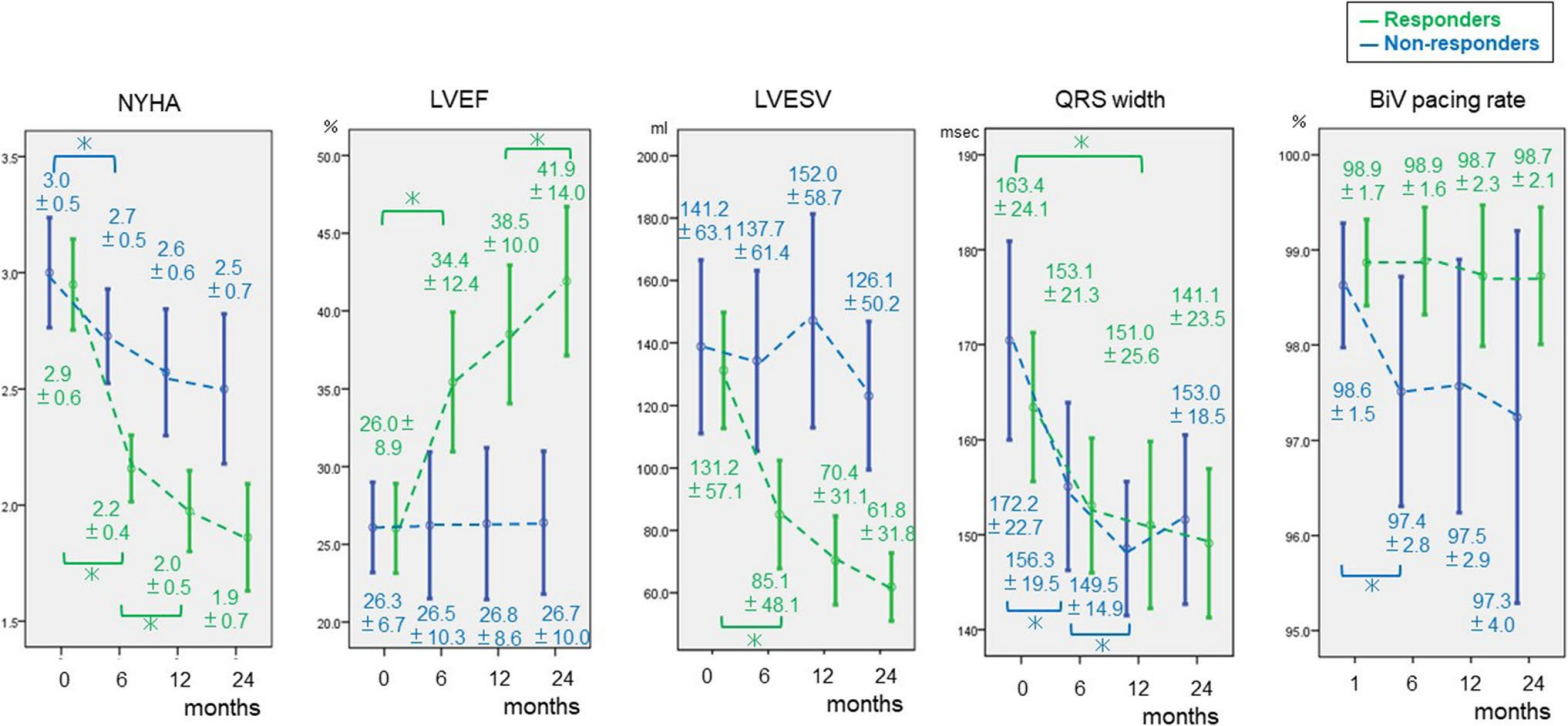
Time course of NYHA classification, LVEF, LVESV, QRS width, and BiV pacing rate in group 1. This shows the time course (at 0 or 1, 6, 12 and 24 months) of the following parameters in group 1, focusing on responders and non-responders. The LVESV of the responders in group 1 was significantly reduced, but the LVESV of the non-responders in group 1 did not change. The QRSw of the non-responders and responders in group 1 decreased significantly. Blue line: non-responders in group 1, green line: responders in group 1,　*: significant difference, *NYHA* New York heart association classification, *LVEF* left ventricular ejection fraction, *LVESV* left ventricular end-systolic volume, *QRSw* QRS width, *BiVpr* Bi-ventricular pacing rate

**Fig. 6:**
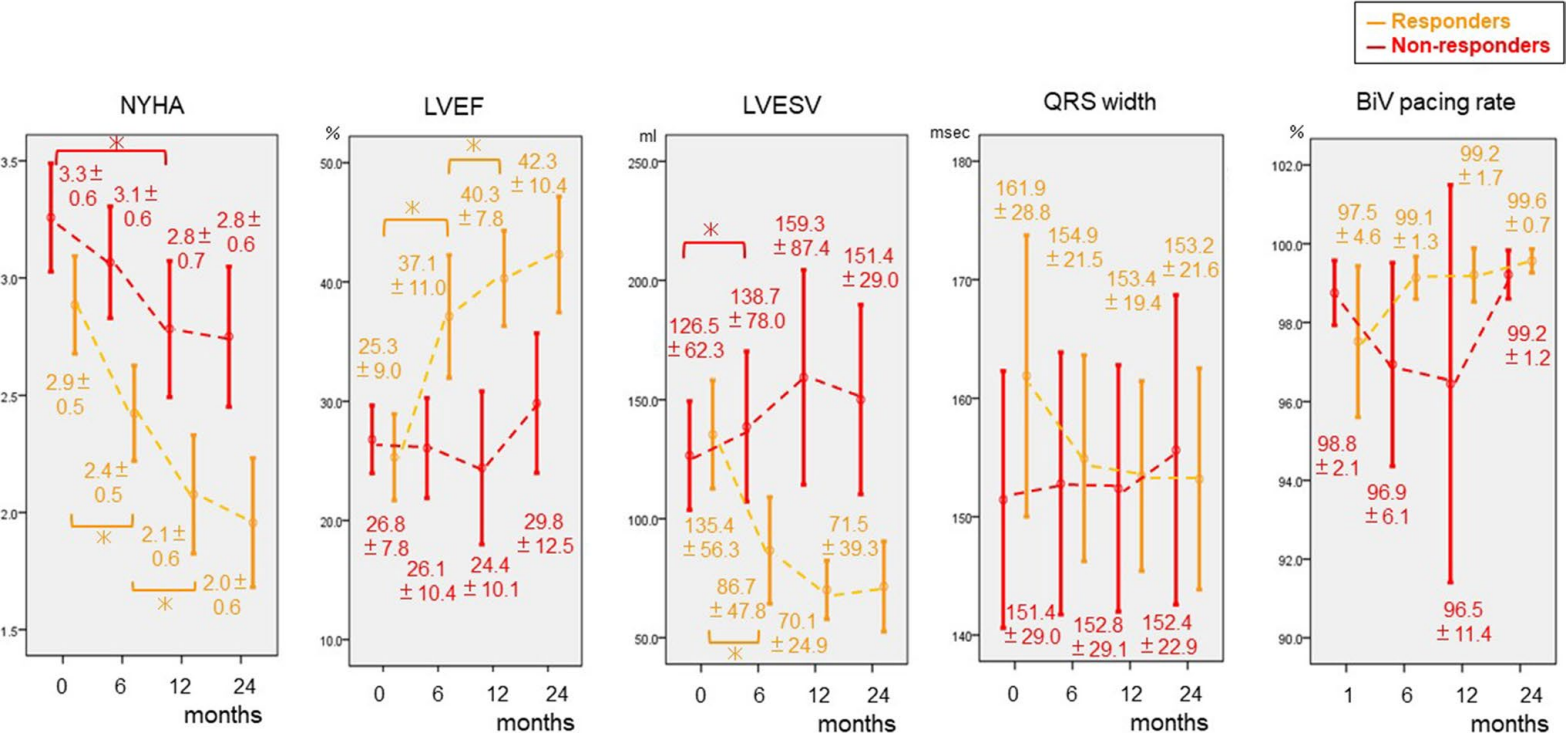
Time course of NYHA classification, LVEF, LVESV, QRS width, and BiV pacing rate in group 2. This shows the time course (at 0 or 1, 6, 12 and 24 months) of the following parameters in group 2, focusing on responders and non-responders. The LVESV of the responders in group 2 was significantly reduced, but the LVESV of the non-responders in group 2 was significantly increased. The QRSw of the non-responders and responders in group 2 did not change significantly. Red line: non-responders in group 1, orange line: responders in group 1,　*: significant difference, Abbreviations are same as Fig. 5

Figures 5 and 6 show the time course of New York Heart Association (NYHA) classification, LVEF, LVESV, QRS width, and BiV pacing rate up to initial 24 months after CRT implantation in group 1 and 2.

The mean NYHA classification of responders in group 1 at 6 months was significantly improved from the initial value (2.9 ± 0.6 to 2.2 ± 0.4, *p* < 0.001), and it was significantly improved at 12 months as well (2.2 ± 0.4 to 2.0 ± 0.5, *p* = 0.006). NYHA classification of non-responders in group 1 also improved significantly at 6 months from the initial value (3.0 ± 0.5 to 2.7 ± 0.5, *p* = 0.01), but no significant change was observed subsequently. The mean LVEF and LVESV of group 1 at 6 months were significantly improved from the initial values (LVEF: 26 ± 8.9 to 34.4 ± 12.4%, *p* < 0.001; LVESV: 131.3 ± 57.1 to 85.1 ± 48.1 ml, *p* < 0.001). Additionally, LVEF at 12 month was significantly improved compared to 6 months (38.5 ± 10.0 to 41.9 ± 14.0%, *p* = 0.03). There were no significant changes in LVEF and LVESV of non-responders.

The mean QRS width of responders in group 1 at 12 months was significantly smaller than the initial value (163.4 ± 24.1 to 151.0 ± 25.6 ms, *p* = 0.05). The QRS width of non-responders decreased significantly at 6 months (172.2 ± 22.7 to 156.3 ± 19.5 ms, *p* = 0.01) from initial value, and that at 12 months also significantly decreased from 6 months (156.3 ± 19.5 to 149.5 ± 14.9 ms, *p* = 0.01). The mean BiV pacing rate of group 1 was over 97%, which was excellent, but in the non-responders, the mean BiV pacing rate at 6 months decreased from the initial value (98.6 ± 1.5 to 97.4 ± 2.8%, *p* = 0.04).

In Fig. 6, the mean NYHA classification at 6 months in responders of group 2 was significantly improved from the initial value (2.9 ± 0.5 to 2.4 ± 0.5, *p* < 0.001), and that of 12 month was significantly improved compared to 6 months (2.4 ± 0.5 to 2.1 ± 0.6, *p* = 0.004). The NYHA classification of non-responders at 12 months improved significantly from the initial value (3.3 ± 0.6 to 2.8 ± 0.7, *p* = 0.03). The mean LVEF and LVESV in responders of group 2 were significantly improved from the initial values (LVEF: 25.3 ± 9.0 to 37.1 ± 11.0%, *p* < 0.001; LVESV: 135.4 ± 56.3 to 86.7 ± 47.8 ml, *p* < 0.001) and the LVEF at 12 month was significantly improved compared to 6 months (37.1 ± 11.0 to 40.3 ± 7.8%, *p* = 0.02). There was no significant change of LVEF in non-responders of group 2, but interestingly, mean LVESV in non-responders of group 2 at 6 months significantly increased from the initial value (126.5 ± 62.3 to 138.7 ± 78.0 ml, *p* = 0.02). The mean QRS width of group 2 did not change significantly in both of responders and non-responders. The mean BiV pacing rate of group 2 was over 96%, and there was no significant change during the follow-up period in both of responders and non-responders.

Table 3 shows that optimizing AVD, defibrillator, and optimal medical therapy (OMT) had a positive impact on the survival rate associated with CRT by the univariate analysis. In contrast, ischemic cardiomyopathy (ICM), VT, non-sustained ventricular tachycardia (NSVT), paroxysmal atrial fibrillation (PAF), low LVEF, diabetes mellitus (DM), chronic kidney disease (CKD), and anemia had a negative impact on survival. Among them, three factors were significantly associated with prognosis by multivariate analysis. The hazard ratio for survival of long-term optimizing AVD was 3.6 (*p* = 0.01), that of OMT was 3.4 (*p* = 0.05), and that of VT/NSVT was 0.2 (*p* = 0.04).

**Table 3:** Predictors of survivor in non-responders for CRT

Predictors	HR	Univariate analysis Range (95% CI)	*P* value
Continuously Opt AVD	3.2	(1.0–10.49)	0.04
NICM	2.1	(0.64–6.95)	0.24
ICM	0.5	(0.14–1.56)	0.24
With defibrillator	1.4	(0.30–6.18)	0.72
VT/NSVT	0.2	(0.07-0.071)	0.01
PAF	0.7	(0.21–2.49)	0.76
LVEF < 30%	0.6	(0.18–1.80)	0.40
DM	0.9	(0.29–3.18)	1.00
CKD	0.6	(0.17–1.76)	0.40
Anemia	0.4	(0.08–1.86)	0.27
OMT	2.0	(0.46–8.91)	0.48

Multivariate analysis			
Predictors	HR	Range	*P* value
		(95% CI)	
Continuously Opt AVD	3.6	(1.33–9.60)	0.01
VT/NSVT	0.3	(0.13–0.94)	0.04
LVEF<30%	0.8	(0.31–2.16)	0.69
CKD	0.7	(0.28–1.71)	0.43
OMT	3.4	(0.97–12.19)	0.05

## Discussion

### Major findings

There were several important findings in this retrospective study, on the prognosis of patients undergoing CRT. First, long-term optimizing AVD with DBAs significantly increased responder and improved the long-term prognosis and HF hospitalization of patients undergoing CRT. Second, long-term optimizing AVD using DBAs significantly improved the prognosis of the non-responders to CRT, but there was no significant difference between the optimizing AVD group and non-optimizing group in the prognosis of the CRT responders. Third, in CRT non-responders, LVESV did not increase and QRS width decreased in optimizing AVD group (group1), however, LVESV increased and QRS width did not show the decrease in the non-optimizing group (group 2) during the initial 24 months. Fourth, optimizing AVD using DBAs and OMT had a significantly positive effect on the prognosis of non-responders to CRT, while VT/NSVT had a significantly negative effect.

### Optimization of AVD for CRT in previous studies

Optimization of AVD using TTE was usually performed after CRT implantation in the early literature on CRT. For example, the MIRACLE study was published in 2002. Optimization of AVD using TTE was performed at discharge, and 6 and 12 months after CRT implantation. It was performed at discharge and 6 months after CRT implantation in the MIRACLE ICD II trial published in 2004 and was performed at discharge and at 3, 6, and 18 months in the CARE HF trial [3, 14]. Optimization of AVD is considered essential to maximize the effect of CRT in large randomized control studies, because it is well known that the best AVD is changed in accordance with LV remodeling. Mullens et al., reported that 47% of the reasons for non-responder to CRT was inadequate optimized AV delay and 32% of the reasons was arrhythmias [6]. Many methods, such as Ritter’s method and methods using LV outflow VTI or ventricular inflow pattern have been reported for optimizing AVD under TTE guidance. However, there is no fixed method of when and how often the AVD should be optimized [15, 16]. Kosmala et al., performed a meta-analysis of the effect of optimal AVD using TEE, on CRT. According to the report, although cardiac function was significantly improved, the quality of life (QOL) and 6-min walking did not improve, and the outcomes were limited. The authors speculated that optimal AV and VV intervals may change over time in response to altered LV loading conditions and reverse remodeling [17].

Recently, several devices with the function to optimize AVD using intracardiac electrocardiogram have been made available, they include QuickOpt (Abbott), SyncAV (Abbott), SmartDelay (Boston Scientific) and aCRT (Medtronic) [9–13]. Ellenbogen et al., reported that the optimal AVD using SmartDelay did not change the responder rate when compared to TTE-guided AVD optimization and fixed AVD; therefore, the effectiveness of optimal AVD using DBAs is still controversial [10]. Another report showed the significance of continuous optimization of AVD throughout life, because cardiac function usually deteriorates with age and optimal AVD can change over time [18]. The study period in previous studies that evaluated the effect of optimal AVD using DBAs was relatively short (6–24 months), and there are no reports that show long-term results. Even in the longest study, the RESPOND CRT trial, which showed the effect of the Respond-CRT (Microport) algorithm, the observation period was 24 months. The study showed a decrease in the incidence rate of HF hospitalization and a tendency towards better survival rates, although it was not statistically significant [13].

Therefore, we investigated the effect of long-term use of DBAs in CRT on patient survival rate in this retrospective study. We found that optimizing AVD using DBAs significantly increased the number of responders and improved their long-term prognosis. Interestingly, optimizing AVD in non-responders significantly improved their prognosis.

### Mechanism of improvement by long-term optimizing AVD

Sub-optimal AVD in patients with HFrEF is known to have a negative effect on hemodynamics. It has also been reported that the more severe HF, the more the PQ interval increases [19]. There have been many reports that AVD optimization for pacemaker in patients who were diagnosed with HFrEF could improve hemodynamics by adjusting LV inflow pattern, eliminating diastolic mitral regurgitation, and improving right and LV cardiac output [20–22]. However, the optimal AVD in patients with HF could change over time, and AVD should be adjusted in accordance with the change in condition.

Scharf et al., focused on the heart rate in CRT and reported that the optimal AVD changed as the heart rate changed [23]. It is thought that the optimal AVD can change, since heart rate changes over the long term in patients with HF depends on the dose of beta blocker (which is the standard treatment for HF), the use of antiarrhythmic drugs, and the status of HF. Furthermore, aCRT, which determines and changes the optimal AVD per minute, improved the composite endpoint of CRT [24].

Additionally, in Fig. 6, non-responders without DBAs as group 2 did not change the mean QRS width and the mean LVESV increased significantly. In contrast, the mean QRS width of non-responders with DBAs in group 1 was reduced and the LVESV of this group did not increase in Fig. 5. Some reports demonstrated a relationship between QRS narrowing and the effectiveness of CRT. SyncAV, relatively new DBAs, has proven to make ECGs narrower significantly [25–27]. According to the results of those reports, it is speculated that long-term DBAs use may promote electrical reverse remodeling and suppress mechanical remodeling by optimizing AVD which is constantly changing as a result of medication and the status of HF and can improve survival even in CRT non-responders, as a result.

Our results of multivariate analysis support the claim that optimal AVD using DBAs and OMT may have interacted.

### Predictors of survival in CRT non-responders.

Despite the very poor prognosis of CRT non-responders, no reports have investigated the factors that contribute to the survival of non-responders for CRT. Our study indicated that the optimizing AVD using DBAs improved the prognosis of CRT non-responders; therefore, we also analyzed other factors that have been generally known to influence the prognosis of patients with HFrEF using multivariate analysis. In addition to optimizing AVD, OMT was a significant predictor of positive impact on survival in non-responders to CRT, while VT/NSVT in comorbidities had a significantly negative impact. It goes without saying that the importance of OMT for HF therapy was also confirmed among the non-responders in this study. According to the guidelines, OMT for HFrEF remains most important, and it is recommended that CRT should be considered after at least three months of OMT [4, 5]. It has been reported that NSVT inhibits LV reverse-remodeling after CRT implantation. On the other hand, CRT in patients with good LV reverse remodeling significantly suppresses appropriate shock of ICD [28–30]. Interestingly, the defibrillator had a lesser effect on survival than the VT/NSVT. Therefore, it is suggested that VT/NSVT may have other effects on the survival rate associated with CRT beside prevention of sudden death. Moreover, ventricular arrhythmia has a significant influence on the BiV pacing rate. Ruwald et al., reported that there was a correlation between the incidence of premature ventricular contraction (PVC) and the CRT responder rate, and PVC reduced the BiV pacing rate [31]. Maintaining a high BiV pacing rate in CRT is thought to be very important in improving the response rate in CRT. Koplan et al., reported that all-cause mortality and HF hospitalization were significantly improved in patients whose BiV pacing rates were maintained at 92% or more [32]. The results of our study indicate that more attention needs to be paid to ventricular arrhythmias in CRT non-responders.

### Clinical implications

The results of this study have several clinical implications. First, this study confirmed the importance of optimizing AVD using DBAs in the long-term, and this idea was also applicable to non-responders to CRT. The mechanism can be improvement of electrical remodeling and suppression of worsening mechanical remodeling. Second, this study emphasized the beneficial effect of OMT and the treatment for VT/NSVT in terms of the prognosis in non-responders to CRT. This could be very meaningful because the prognosis of non-responders to CRT is usually considered poor.

### Study limitation

Our study has several limitations. This was a single-center retrospective study with a relatively small sample size. Therefore, further studies are needed to determine the benefit of long-term optimizing AVD. The two groups in this study have not been rigorously coordinated. Specifically, the ECG width, distribution of device manufacture and rate of amiodarone use between the two groups were different. These differences in patients’ characteristics may have affected outcomes. The environment around CRT practice and cardiac implantable device itself changed dramatically during this study, specifically, the development of quadri-polar leads, multi-point pacing system and remote monitoring system. The effect of those changes has not been assessed. Additionally, medication for HF has changed significantly in recent years.

Unfortunately, it was impossible to investigate medication changes because of the small information of the device clinic. ARNI and SGLT2 inhibitor, which are new agents for HF, were not covered by Japanese insurance during this study period, so the impact of these agents was considered to be small. However, unexamined change of medication is also limitation of this study. Furthermore, this multivariate analysis in small number studies may be inadequate because a sufficient number of parameters cannot be selected.

## Conclusion

Long-term, optimizing AVD using DBAs improved the survival rate in patients undergoing CRT and improved the prognosis of CRT non-responders, as well.

## Supplementary Information

Below is the link to the electronic supplementary material.

Supplementary file1 (JPG 76 KB)

Supplementary file2 (JPG 107 KB)

Supplementary file3 (JPG 150 KB)

Supplementary file4 (JPG 147 KB)

Supplementary file5 (JPG 82 KB)

Supplementary file6 (JPG 89 KB)
