# Supplementary figures and images for: Impact of long-term optimizing atrioventricular delay using device-based algorithms on cardiac resynchronization therapy
Source: Heart Vessels. 2022 Sep 29;38(2):216–27. doi: 10.1007/s00380-022-02162-4 (PMC9816250; doi:10.1007/s00380-022-02162-4)

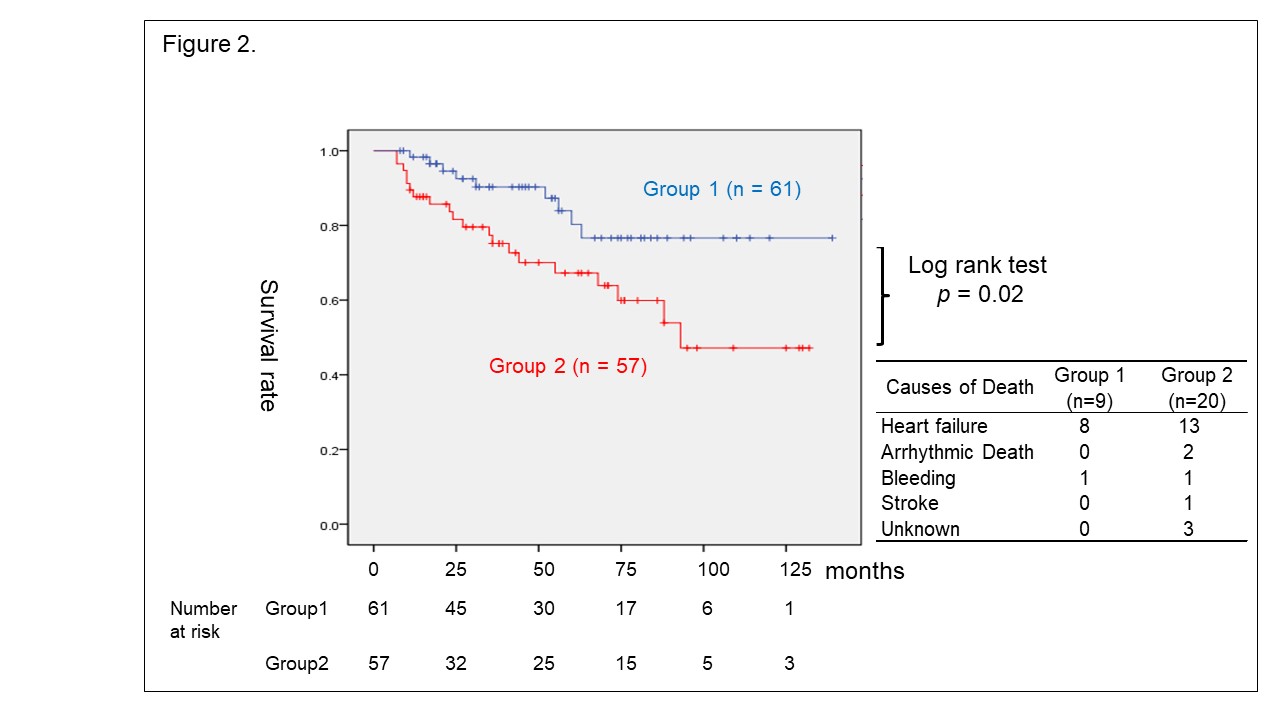

Supplement: Supplementary file 1 — Supplementary file1 (JPG 76 KB) [file 380_2022_2162_MOESM1_ESM.jpg]

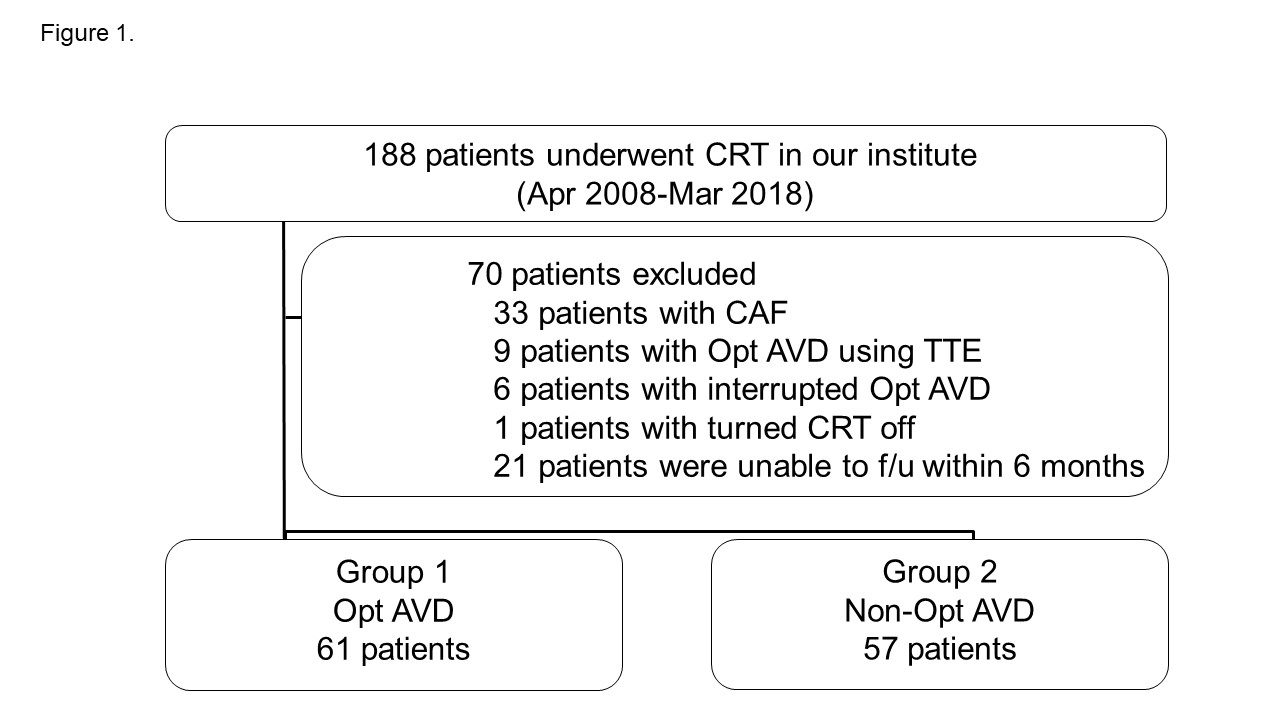

Supplement: Supplementary file 2 — Supplementary file2 (JPG 107 KB) [file 380_2022_2162_MOESM2_ESM.jpg]

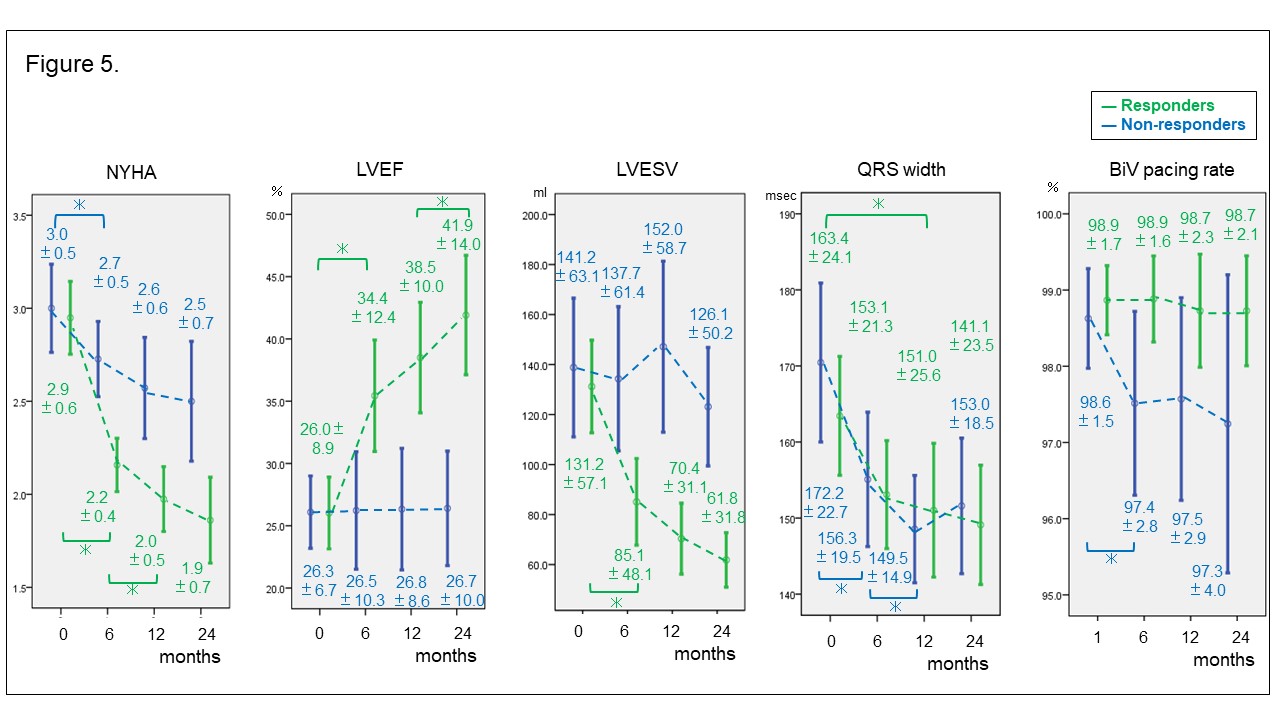

Supplement: Supplementary file 3 — Supplementary file3 (JPG 150 KB) [file 380_2022_2162_MOESM3_ESM.jpg]

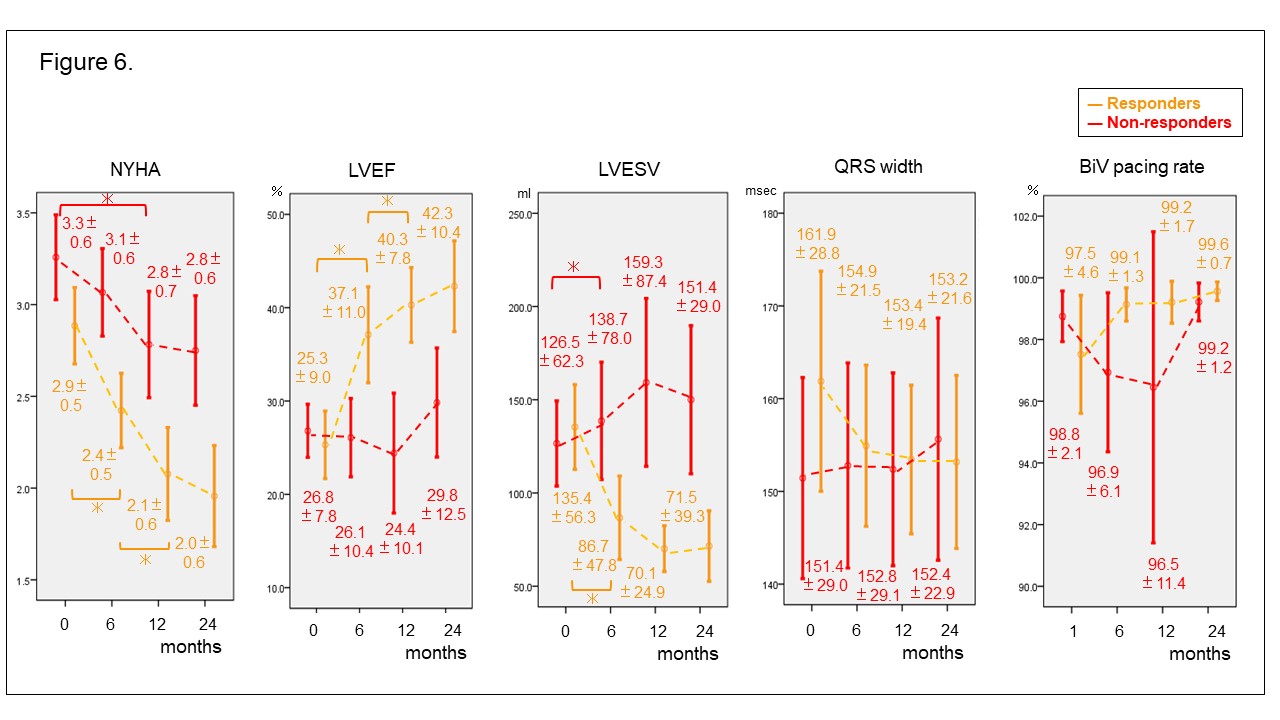

Supplement: Supplementary file 4 — Supplementary file4 (JPG 147 KB) [file 380_2022_2162_MOESM4_ESM.jpg]

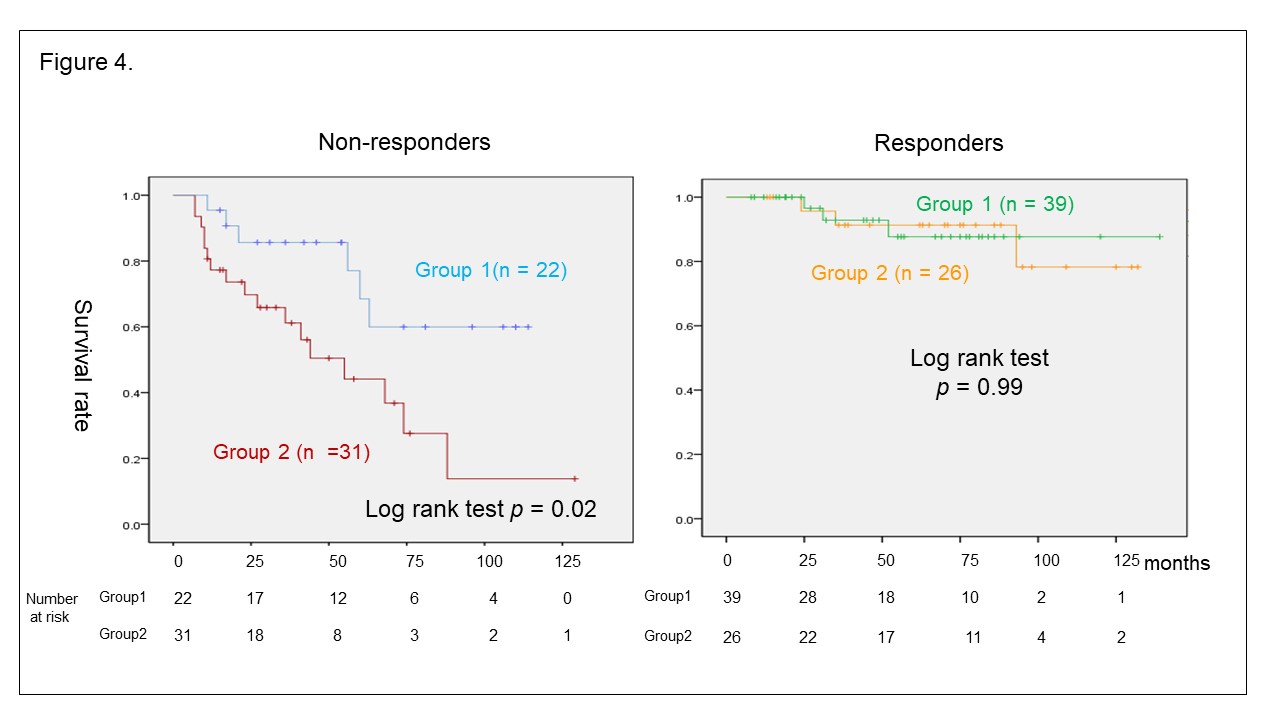

Supplement: Supplementary file 5 — Supplementary file5 (JPG 82 KB) [file 380_2022_2162_MOESM5_ESM.jpg]

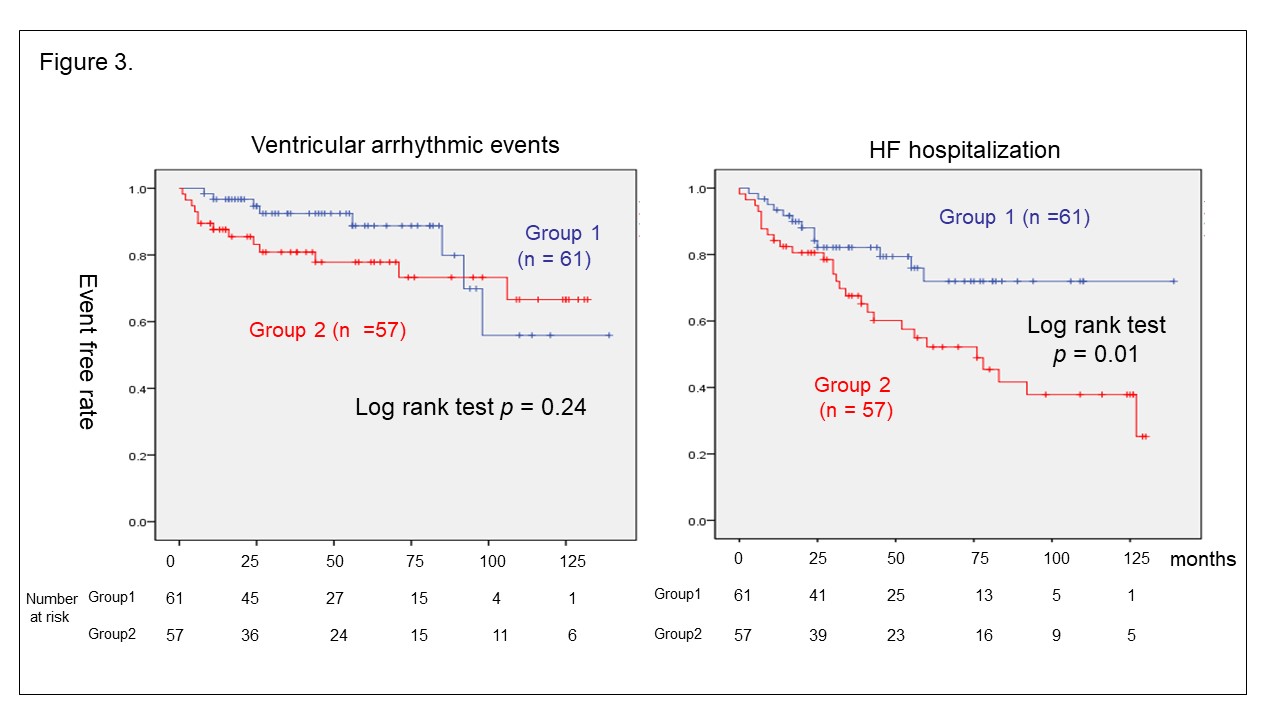

Supplement: Supplementary file 6 — Supplementary file6 (JPG 89 KB) [file 380_2022_2162_MOESM6_ESM.jpg]
